# Research hotspots and prospects on the correlation between subchondral bone and stem cells: bibliometrics and visual analysis

**DOI:** 10.3389/fsurg.2026.1781739

**Published:** 2026-04-13

**Authors:** Weibin Du, Haolin You, Ying Fang, Zhenwei Wang, Chengying Lin, Yanghua Tang, Fuxiang Shen, Guoping Cao, Gang Qu

**Affiliations:** 1 Research Institute of Orthopedics, the Jiangnan Hospital affiliated to Zhejiang Chinese Medical University, Hangzhou, Zhejiang, China; 2 Hangzhou Xiaoshan Hospital of Traditional Chinese Medicine, Hangzhou, Zhejiang, China

**Keywords:** bibliometrics, correlation, stem cells, subchondral bone, visual analysis

## Abstract

**Background:**

While the repair and regeneration of cartilage injuries remain a significant research challenge, the relationship between subchondral bone and stem cells has emerged as a new focus. However, a bibliometric analysis of the research trends in this specific area is currently lacking.

**Methods:**

We searched Web of Science Core Collection (WOSCC), Scopus, and PubMed for all relevant literature on the subchondral bone-stem cell relationship from database inception to September 3, 2025. CiteSpace was used to visualize annual publication counts, authors, institutions, countries, co-citations (authors, journals, references), and keywords.

**Results:**

A total of 1,267 publications were included. Research on the subchondral bone-stem cell relationship is an emerging focus. The most prolific author was Zhang Wei from China (15 publications). The institution with the most publications was the Chinese Academy of Sciences (52 publications), which also had the highest centrality (0.19). The most highly-cited author was ZHEN GH. AM J Sport Med was the most highly-cited journal and had the highest centrality (0.31). Keyword analysis showed “mesenchymal stem cells” as the most frequent term, while “cartilage” had the highest keyword centrality (0.27). The top 10 largest keyword clusters were #0 monosodium iodoacetate, #1 chitosan, #2 osteochondral defects, #3 scatter factor, #4 silk fibroin, #5 tissue engineering, #6 edetic acid, #7 microgels, #8 knee, and #9 cartilage repair, which were emerging research fronts.

**Conclusion:**

Research on the subchondral bone-stem cell relationship is an emerging focus. Consistent hotspots include cartilage and bone repair, cell therapy and tissue engineering, animal models and experiments, as well as transplantation and *in vitro*- studies. Simultaneously, advances in technology and deeper research are establishing *in vivo* studies, real-time monitoring techniques, and the investigation of specific factors and signaling pathways as new research hotspots.

## 1 Introduction

Articular cartilage injury, one of the most common musculoskeletal disorders, is often caused by acute trauma, chronic overuse, or degeneration. Its repair and regeneration remain a major research focus and significant challenge in the relevant fields (1–3). Due to its unique avascular structure and complex biological characteristics, articular cartilage exhibits limited self-repair capacity after injury (4, 5). Anatomically, cartilage is firmly integrated with the subchondral bone, forming a functional osteochondral unit. Articular cartilage is the elastic, load-bearing tissue covering the bony articular surfaces. Subchondral bone, located at the bone-cartilage interface, consists of a superficial cortical endplate and underlying trabecular structures of cancellous bone. The subchondral bone absorbs shock, bears mechanical loads, and connects bone to cartilage (6–8). Articular cartilage, as an avascular tissue, possesses an extremely limited regenerative capacity. Structural and functional integrity following injury is rarely restored, predisposing to degenerative pathologies such as osteoarthritis (OA) (9). Research indicates that metabolic imbalances in trace elements—including iron (Fe), copper (Cu), and zinc (Zn)—are closely associated with OA onset and progression. Excess iron promotes chondrocyte apoptosis and upregulates matrix metalloproteinase (MMP)-3/13 expression, while activating the mTORC1-p70 S6 kinase/4E-BP1 signaling pathway to exacerbate synovial inflammatory responses, thereby accelerating the progression of joint degeneration (10). The role of the subchondral region is receiving growing attention in articular cartilage injury, osteoarthritis pathogenesis, and treatment. Knee osteoarthritis alone affects approximately 240 million people globally, posing a substantial socioeconomic burden (11, 12). Consequently, numerous therapies targeting the subchondral region are being explored to treat cartilage injuries and osteoarthritis (13–18).

Clinically, subchondral bone defects are often treated with bone cement or autologous bone grafting to fill the lesion and restore structural support for cartilage (19, 20). However, bone cement can cause thermal damage, impairing the biomechanical conduction between cartilage and subchondral bone. Autologous bone grafting is associated with limitations such as donor-site morbidity, risk of infection, and limited graft availability. The microfracture technique creates drill holes penetrating the subchondral bone, allowing marrow-derived blood and mesenchymal stem cells to access the injury site and promote cartilage repair (21, 22). Recently, tissue engineering approaches utilizing bone marrow mesenchymal stem cells (23), cartilage stem/progenitor cells (24), or synovial mesenchymal stem cells (25) in combination with biocompatible scaffolds have been increasingly used to repair cartilage and subchondral bone defects (26, 27). Although a substantial body of empirical studies has established the interrelationship between stem cells and subchondral bone repair while synthesizing specific advancements, a comprehensive synthesis of research advancements remains lacking. Therefore, a systematic bibliometric analysis encompassing publications, countries, institutions, journals, authors, and keywords is urgently required to elucidate the evolving research landscape and knowledge architecture in this domain.

This study employed CiteSpace (28, 29) and VOSviewer (30) to analyze current research on the subchondral bone-stem cell relationship. We comprehensively reviewed relevant literature from WOSCC, Scopus, and PubMed, created visualized knowledge maps, and identified research hotspots and trends to inform future studies. This study employed a bibliometric methodology to characterize research outputs at the national, institutional, and authorial levels, analyze highly cited publications, and map thematic evolution and research frontiers. By systematically mapping the evolutionary trajectories of stem cell and subchondral bone research, this analysis aims to identify emerging research hotspots and provide valuable insights for guiding both clinical applications and fundamental investigations in this field.

## 2 Materials and methods

### 2.1 Data sources and search strategy

The Web of Science Core Collection (WoSCC), Scopus, and PubMed databases were selected as data sources. The search period spanned from database inception to September 3, 2025, with all records restricted to English-language publications. The search strategies were as follows: WoSCC: TS = (“subchondral bone*”) AND (“stem cell*”); Scopus: TITLE-ABS-KEY(“subchondral bone*”) AND TITLE-ABS-KEY(“stem cell*”); PubMed: (“subchondral bone*"[MeSH Terms] OR “subchondral bone*”[Title/Abstract]) AND (“stem cell*”[MeSH Terms] OR “stem cell*”[Title/Abstract]). Document types were limited to “Article,” excluding reviews, early access publications, and book chapters. Deduplication and Record Processing: Python software was employed to convert Scopus and PubMed datasets into WoS-compatible plain-text format. These datasets were then merged with the WoS-exported data and deduplicated. A final dataset of 1,267 publications was included for analysis.

### 2.2 Methods

**Figure 1:**
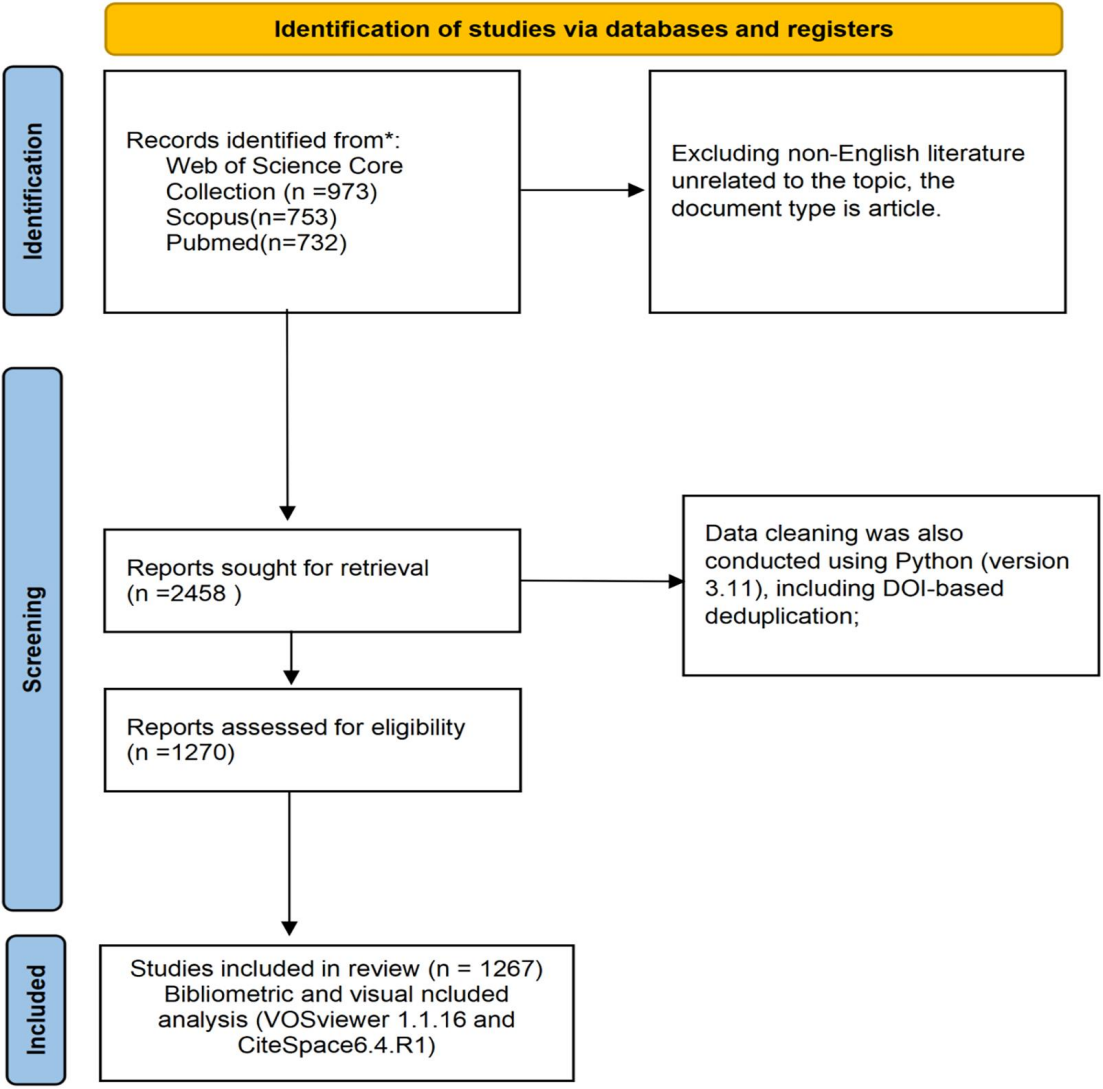
Literature screening and analysis flowchart.

**Figure 2:**
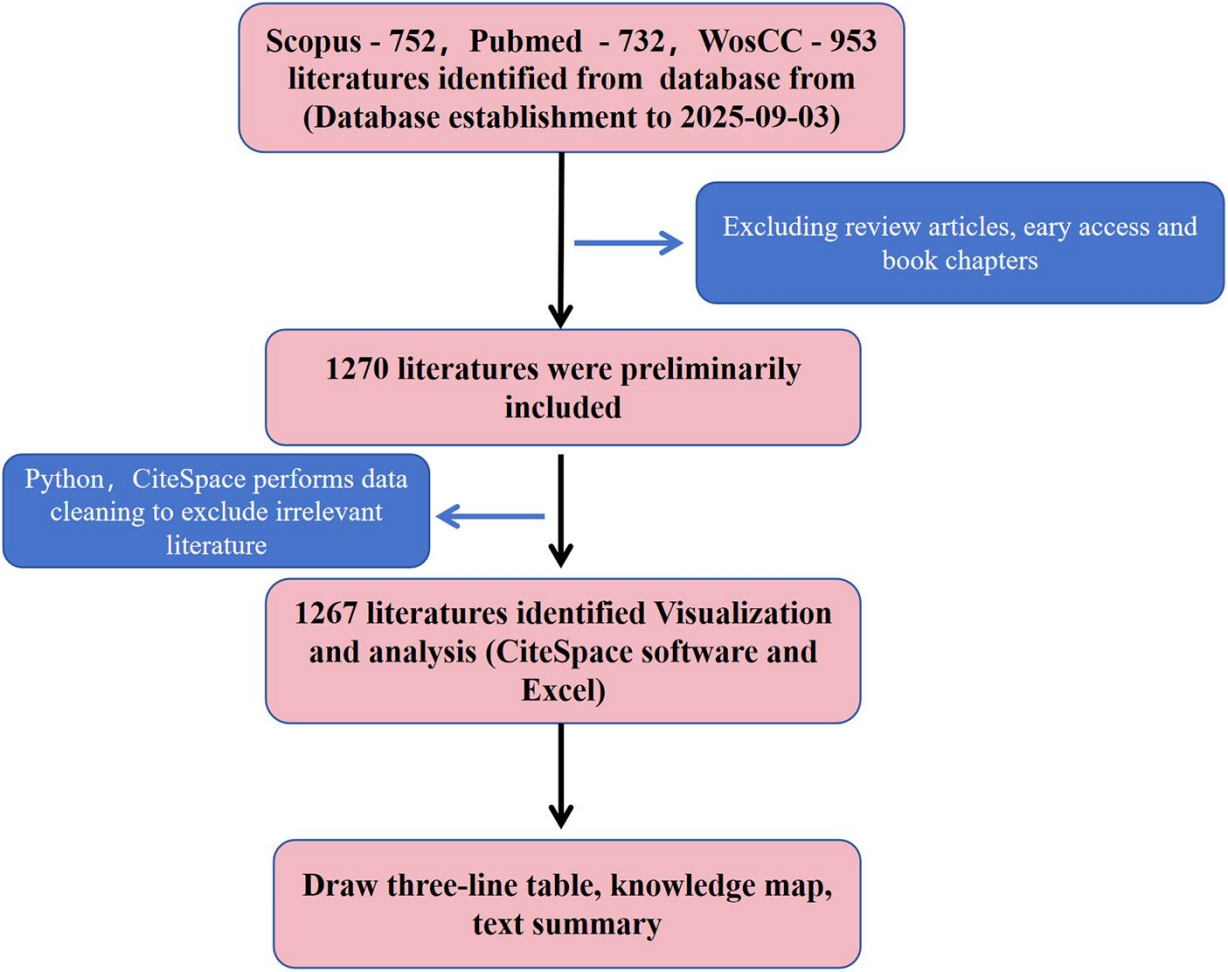
Literature screening and analysis flowchart.

The initial search yielded a total of 2458 records (WOSCC: 973 records; Scopus: 753 records; PubMed: 732 records). Two independent reviewers (A and B) independently screened all retrieved records for relevance, with disagreements resolved by a third reviewer (C). After excluding irrelevant studies, bibliographic data were downloaded in plain text and CSV formats, including full records and cited references. Python (version 3.11) was used to convert Scopus CSV files and PubMed TXT files into a unified plain text format consistent with the WOSCC full record and cited references structure. Data cleaning was also conducted using Python (version 3.11), including DOI-based deduplication; removal of retracted publications; exclusion of records with “[Anonymous]” in the author field; elimination of virtual institutions such as the Egyptian Knowledge Bank (EKB); and standardization and merging of duplicated institution names. Ultimately, 1267 publications were retained for subsequent bibliometric analysis. The literature selection process is illustrated in Figures 1, 2.

A total of 1,270 records were renamed as “download_XXX.txt” and stored in an “input” folder, then imported into CiteSpace (version 6.4.R1) and VOSviewer for bibliometric analysis. The software successfully processed 1,267 records. In CiteSpace, the following settings were applied: Node types: Author, Institution, Country, Cited Author, Cited Journal, Cited Reference, and Keyword.Selection criteria: The top 50 most cited or frequently occurring items per time slice were retained. Pruning algorithms: “Pathfinder,” “Pruning sliced networks,” and “Pruning the merged network” were employed to simplify network structures and emphasize core connections. Thresholds and parameters: (LRF=3), year of review (LBY=5), e (*N* = 1), time span (2012–2022), year per slice (1), link (strength: cosine, range: slice), selection criteria (g-index: k = 25), and minimum duration (key words:MD = 2; Reference:MD = 2 reference). link (strength: cosine, range: slice), selection criteria (g-index:k = 25), and minimum duration (key words:MD = 2; Reference:MD = 2 reference). In VOSviewer, the following parameters were used: Analysis: Normalization method=Association strength. Layout: Attraction=2, Repulsion=1 Clustering: Resolution=1, Minimum cluster size=1, Orientation: Rotation=90 degrees. Cluster stability was assessed through sensitivity analysis. Based on these configurations, visualizations and knowledge maps were generated. As the dataset contained no personally identifiable or patient-level information, ethical approval was not required.

### 2.3 Statistical analysis

Microsoft Office Excel 2021 was used for data management and analysis of annual publication trends and proportions. Bibliometric and visual analyses were performed using CiteSpace and VOSviewer. Specifically, CiteSpace version 6.4.R1 was employed to conduct collaborative network analyses of countries/regions and institutions, author collaboration, cited journals, cited authors, cited references, and keyword co-occurrence. VOSviewer was used for mapping collaborations among countries, institutions, and authors.

In the CiteSpace visualization maps, a purple ring around a node indicates high betweenness centrality, identifying that node as a pivotal or hub entity in the field. In VOSviewer visualizations, nodes sharing the same color belong to the same research cluster or thematic direction. Connections between nodes represent co-occurrence or co-citation relationships, with thicker lines indicating stronger associations.

## 3 Results

### 3.1 Annual publication analysis

**Figure 3:**
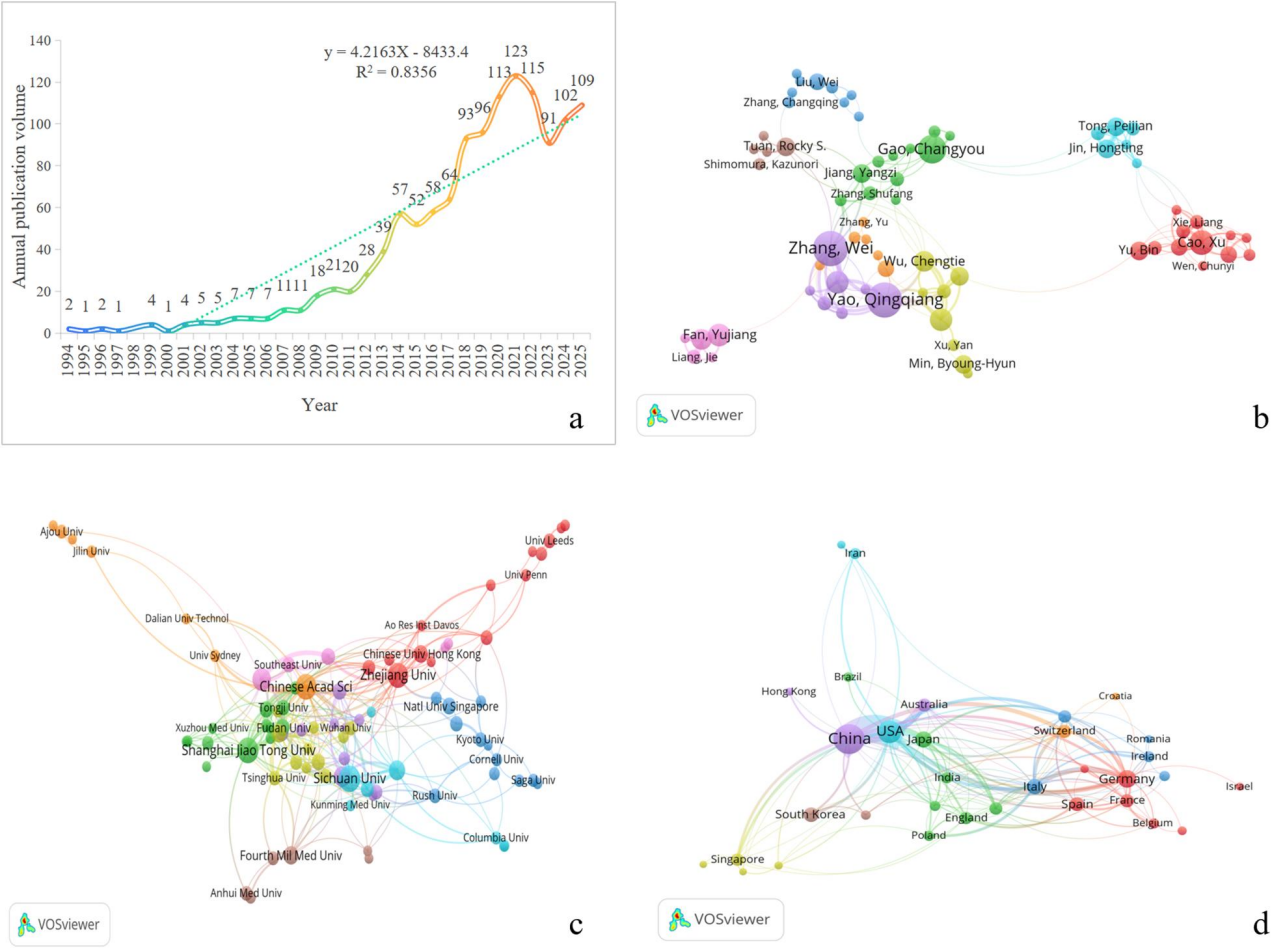
**(a)**: The annual number of publications. **(b)**: Knowledge map of author collaboration. **(c)**: Knowledge map of institution collaboration. **(d)**: Knowledge map of country collaboration.

Trend charts of annual and cumulative publications were plotted using Excel (Figure 3a). From 1994 to 2025, the annual number of publications in this field showed a steady increasing trend overall, peaking at 123 publications in 2021. During this period, research on the relationship between subchondral bone and stem cells demonstrated significant linear growth (R^2^ = 0.8356). Publications from 2025 were excluded from the trend analysis due to incomplete data, but projections based on the linear function (Y = 4.2163X-8433.4) indicate a continued upward trend. These findings collectively indicate that research on the subchondral bone-stem cell relationship is an emerging focus, and its research interest continues to grow.

### 3.2 Analysis of authors, institutions, and countries/regions

The author collaboration network was constructed using VOSviewer (Figure 3b), revealing several prominent research teams centered around key authors including Zhang Wei, Yao Qingqiang, Gao Changyou, Tong Peijian, Cao Xu, and Tuan Rocky S. Among the top ten most prolific authors in terms of publication count between 1994 and 2025 (Table 1), three authors each published more than 10 papers: Zhang Wei (15 publications, 1.18%), Yao Qingqiang (14 publications, 1.10%), and Gao Changyou (10 publications, 0.79%). The majority of these top ten authors are affiliated with institutions in China, indicating that China represents the most concentrated hub of research activity in this field.

**Table 1:** The top 10 authors by number of publication.

Number	Author	Article counts	Article counts	Country	Total citations	H-index
1	Zhang, Wei	17	1.18	China	54	15
2	Yao, Qingqiang	14	1.10	China	67	15
3	Gao, Changyou	10	0.79	China	25	14
4	Mikos, Antonios G	9	0.71	USA	33	11
5	Cao, Xu	8	0.63	China	40	14
6	Fan, Yujiang	8	0.63	China	27	8
7	Wu, Chengtie	8	0.63	China	34	9
8	Chen, Jialin	8	0.63	China	43	13
9	Jones, Elena	8	0.63	England	7	7
10	Zhang, Xingdong	6	0.47	China	24	10

We constructed an institutional collaboration network using VOSviewer (Figure 3c). Analysis based on color clustering and link strength revealed distinct collaborative groups centered around Sichuan University, Shanghai University, Zhejiang University, Chinese Academy of Sciences, Peking University, Fourth Military Medical University, Nanjing Medical University, National University of Singapore, and Chinese Academy of Medical Sciences. Notably, the Chinese Academy of Sciences maintains particularly strong collaborative ties with Zhejiang University and Nanjing Medical University.

Further analysis of high-output institutions (Table 2) showed that the Chinese Academy of Sciences ranked first in total publication output and exhibited the highest centrality score (0.19), underscoring its role as a pivotal hub within the collaboration network. Remarkably, nine of the top ten most productive institutions are based in China, collectively accounting for 22% of the total publications—highlighting China's dominant position and robust internal collaboration capacity in this research field. The high productivity of Chinese Academy of Sciences-affiliated authors, such as Zhang Wei and Yao Qingqiang, further reflects its effective team-based research model and sustained scholarly output.

**Table 2:** The top 10 institutions by number of publication.

Number	Institution	Article counts	Centrality	Country	Total citations
1	Chinese Academy of Sciences	52	0.19	China	73
2	Sichuan University	50	0.02	China	29
3	Shanghai Jiao Tong University	45	0.03	China	38
4	Zhejiang University	40	0.17	China	29
5	Nanjing Medical University	31	0.03	China	38
6	Southern Medical University - China	24	0.03	China	17
7	Chinese People's Liberation Army General Hospital	20	0.08	China	21
8	Peking University	20	0.07	China	32
9	Johns Hopkins University	19	0.09	USA	34
10	Chinese University of Hong Kong	18	0.05	China	22

We used VOSviewer software to construct a country/region collaboration network diagram, consolidating synonymous nodes (such as the United States and the USA) (Figure 3d). Analysis based on color clustering and link strength has revealed international academic clusters represented by countries including China, the United States, Japan, Germany, and others. Among these, China, the United States, Australia, as well as the United States and Japan, Germany exhibit particularly close international collaborations.

Among the top ten most productive countries/regions in terms of publication output (Table 3), China (573 publications, 45.22%) and the United States (261 publications, 20.59%) each published more than 100 papers. Although the U.S. produced only about half as many publications as China, its betweenness centrality was over nine times higher, indicating a more central position within the global research network. Japan ranked fifth with 69 publications but exhibited the highest betweenness centrality (0.70), underscoring its role as the most pivotal hub in the international collaboration network.

**Table 3:** The top 10 countries by number of publication.

Number	Country	Counts	Centrality	Total citations
1	China	573	0.04	123
2	USA	261	0.39	141
3	Germany	92	0.22	67
4	Japan	69	0.70	39
5	Italy	61	0.23	51
6	South Korea	47	0.04	14
7	Spain	43	0.18	18
8	Australia	36	0.17	31
9	Switzerland	32	0.11	33
10	France	32	0.09	28

In the country collaboration map, the nodes for the United States and Japan appear lighter in color—reflecting their relatively lower total publication volumes compared to China-yet their dense interconnections and high betweenness centrality demonstrate that they serve as critical structural bridges, facilitating knowledge exchange among geographically and institutionally dispersed research communities. This pattern highlights that global collaboration in the field of subchondral bone and stem cell research is predominantly concentrated among high-income countries with mature scientific infrastructures.

In contrast, China leads globally in research output (573 publications, accounting for 45.22% of the world total), demonstrating substantial scientific capacity. However, it exhibits a very low betweenness centrality and a relatively limited international collaboration network—primarily confined to partnerships with the United States and Australia. This pattern suggests that Chinese research in this field remains largely driven by domestic priorities and has not yet been fully integrated into the global innovation ecosystem, highlighting significant room for improvement in international knowledge dissemination and cross-border coordination of research resources.

### 3.3 Analysis of cited authors, cited journals, and cited references

**Figure 4:**
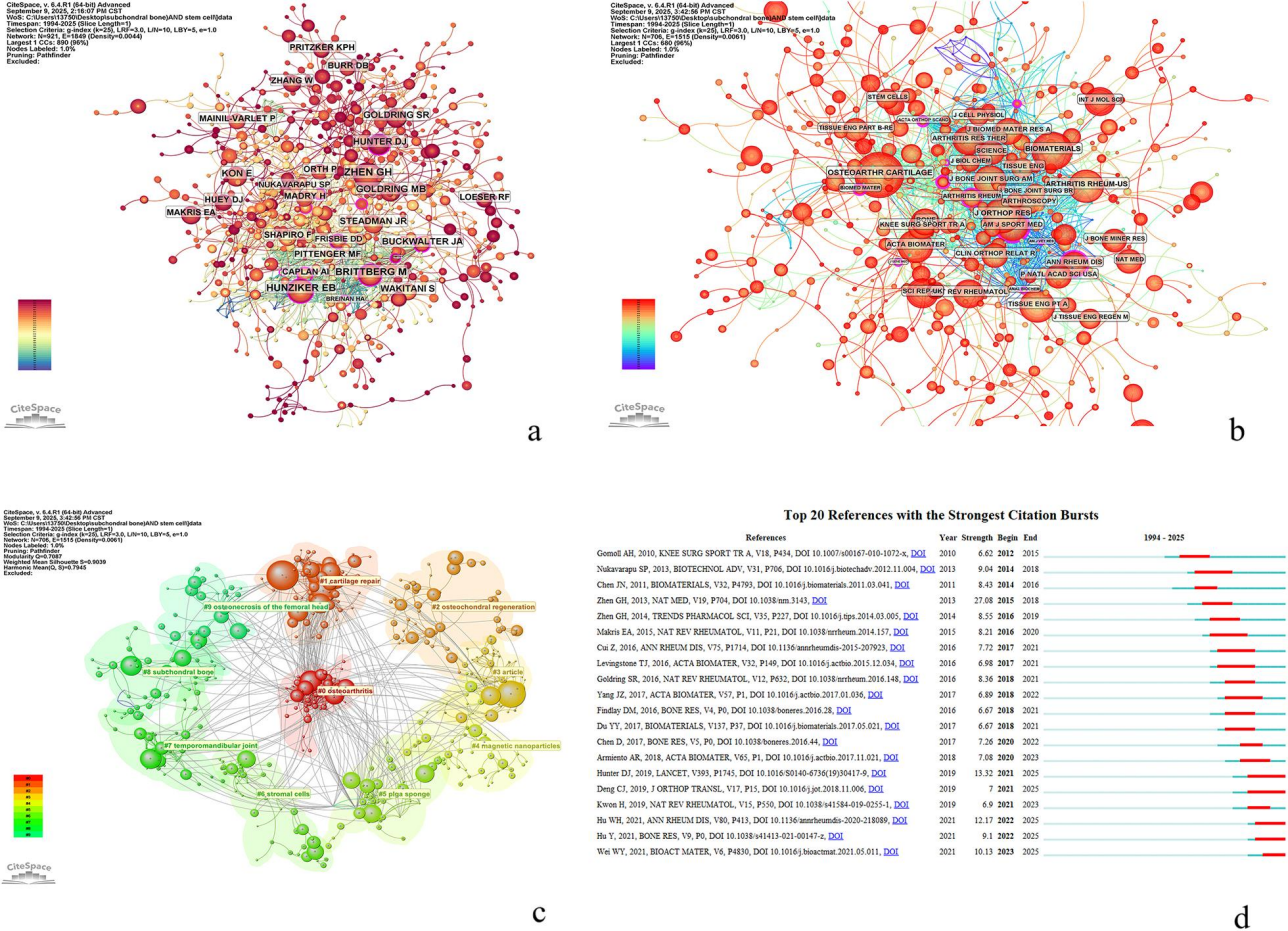
**(a)**: Knowledge map of co-cited author. **(b, c)**: Knowledge map of Journal clusters. **(d)**: Top 20 references with the strongest citation bursts.

Analysis of cited authors generated a co-citation network of 921 nodes and 1,849 links (Figure 4a). Among the top 10 most-cited authors (Table 4), three were cited more than 100 times: ZHEN GH (155 citations), HUNZIKER EB (142 citations), and BRITTBERG M (132 citations). The authors with the highest centrality were BRITTBERG M and BUCKWALTER JA, indicating their pivotal role in the co-citation network. Further examination of structural importance within the cited author network revealed that BRITTBERG M and BUCKWALTER JA exhibited the highest centrality (both 0.17), indicating their pivotal roles as knowledge brokers in integrating domain-specific advancements. A total of 127 authors with ≥3 publications were identified as the core contributor cohort. For collaboration network construction, 89 isolated nodes lacking inter-author collaborations were excluded, resulting in a substantive network of 38 authors (Figure 4a). The five authors with highest total link strength were Wei Zhang (total link strength=12), Yao Qingqiang (10), Gao Changyou(8), Chen Jialin (7), and Fan Yujiang (7), demonstrating their dual prominence in both productivity and collaborative centrality. Notably, Wei Zhang and Qingqiang Yao, both affiliated with the Chinese Academy of Sciences, formed critical collaborative linkages underpinning the institution's leadership in this field. Among high-output authors (non-citation-based metrics), Wei Zhang (15 publications) and Qingqiang Yao (14 publications) emerged as the most prolific contributors, signifying active research teams driving frontier developments in subchondral bone-stem cell interactions.

**Table 4:** The top 10 highly cited authors.

Serial number	Cited author	Count	Centrality	Year
1	ZHEN GH	155	0.03	2015
2	HUNZIKER EB	142	0.13	1999
3	BRITTBERG M	132	0.17	1999
4	GOLDRING MB	97	0.09	2009
5	KON E	96	0.04	2012
6	WAKITANI S	89	0.13	2002
7	STEADMAN JR	86	0.05	2001
8	HUNTER DJ	84	0.02	2015
9	BUCKWALTER JA	81	0.17	1999
10	GOLDRING SR	78	0.03	2016

The cited journal co-citation network consisted of 706 nodes and 1,515 links (Figure 4b). Among the top 10 most-cited journals (Table 5), two journals exceeded 500 citations: Osteoarthritis and Cartilage (727 citations) and Biomaterials (580 citations). Am J Sport Med demonstrated the highest centrality (0.31), indicating its significant influence across the research network. Analysis of the cited journal clustering map (Figure 4b) revealed that both Osteoarthr Cartilage and Am J Sport Med belong to the #1 cartilage repair cluster, focusing on cartilage as the primary research subject and emphasizing cartilage repair techniques. In contrast, Biomaterials was grouped in the #3 article cluster, which centers on studies utilizing non-human biological models (e.g., animal models) to investigate related conditions such as osteoarthritis and substances like hyaluronic acid, employing controlled study designs to evaluate therapeutic outcomes and explore disease mechanisms. Analysis of co-cited journals (Figures 4b, c) reveals key trends in international research on cartilage repair. The field is structured around two core journals: Osteoarthritis and Cartilage and Biomaterials. The former anchors Cluster #1 (“cartilage repair”), emphasizing tissue regeneration and functional restoration, while the latter belongs to Cluster #3 (“animal model–based OA research”), focusing on therapeutic evaluation and mechanistic exploration using non-human models. Notably, although The American Journal of Sports Medicine has a modest publication volume, it exhibits the highest centrality (0.31), underscoring its pivotal role in bridging sports medicine, injury repair, and regenerative strategies. Collectively, the journal network reflects an evolving paradigm characterized by deep integration of basic mechanisms, biomaterials science, and clinical translation—shifting from single-modality interventions toward multimodal, synergistic approaches that combine cells, scaffolds, and microenvironmental regulation. This underscores the increasing interdisciplinarity and translational orientation of global research in cartilage repair.

**Table 5:** The top 10 highly cited journals.

Serial number	Cited journal	Count	Centrality	Year
1	Osteoarthr Cartilage	727	0.01	1999
2	Biomaterials	580	0.04	1999
3	J Orthop Res	439	0	1999
4	Arthritis Rheum-Us	419	0.03	1999
5	J Bone Joint Surg Am	386	0	1999
6	Acta Biomater	358	0.08	2009
7	Am J Sport Med	356	0.31	1999
8	Tissue Eng Pt A	344	0	2010
9	Arthritis Res Ther	333	0	2005
10	Nat Rev Rheumatol	321	0	2012

Analysis of cited references yielded a citation burst visualization of cartilage and stem cell literature (Figure 4c). Among the top 10 most-cited references (Table 6), the top-cited reference was authored by Zhen GH (64 citations), which is consistent with the analysis of cited authors. The burst characteristics of these cited references—including burst strength and duration—are clearly discernible. The top five publications with the strongest citation bursts are, in order: Zhen GH (2013) (strength=27.08, 2015–2018), Chen JN (2011) (strength=8.43, 2014–2016), Gomoll AH (2010) (strength=6.62, 2012–2015), Makris EA (2015) (strength=8.21, 2016–2020), and Cui Z (2016) (strength=7.72, 2017–2021), indicating their substantial influence on the field during specific periods. Further analysis reveals that the 2013 study by Zhen GH, published in Nature Medicine, garnered widespread attention due to its proposal of a novel stem cell–based therapeutic strategy; its four-year burst period underscores its status as a landmark contribution. More recently, studies such as those by Hu WH (2021) and Wei WY (2021) began exhibiting citation bursts from 2022 onward, signaling an emerging research focus on the integration of stem cells with biomaterials. Overall, burst detection results show that pivotal publications are concentrated between 2010 and 2025, reflecting a clear evolutionary trajectory from fundamental mechanistic investigations toward clinical translation.

**Table 6:** The top 10 highly cited literatures.

Serial number	Cited literature	Count	Centrality	Year
1	Zhen GH, 2013, NAT MED, V19, P704, DOI 10.1038/nm.3143	64	0.01	2013
2	Hunter DJ, 2019, LANCET, V393, P1745, DOI 10.1016/S0140-6736 (19)30417-9	30	0.01	2019
3	Hu WH, 2021, ANN RHEUM DIS, V80, P413, DOI 10.1136/annrheumdis-2020-218089	28	0.05	2021
4	Nukavarapu SP, 2013, BIOTECHNOL ADV, V31, P706, DOI 10.1016/j.biotechadv.2012.11.004	23	0.13	2013
5	Makris EA, 2015, NAT REV RHEUMATOL, V11, P21, DOI 10.1038/nrrheum.2014.157	22	0.03	2015
6	Cui Z, 2016, ANN RHEUM DIS, V75, P1714, DOI 10.1136/annrheumdis-2015-207923	21	0.01	2016
7	Hu Y, 2021, BONE RES, V9, P0, DOI 10.1038/s41413-021-00147-z	21	0	2021
8	Yang JZ, 2017, ACTA BIOMATER, V57, P1, DOI 10.1016/j.actbio.2017.01.036	20	0.11	2017
9	Goldring SR, 2016, NAT REV RHEUMATOL, V12, P632, DOI 10.1038/nrrheum.2016.148	20	0.03	2016
10	Wei WY, 2021, BIOACT MATER, V6, P4830, DOI 10.1016/j.bioactmat.2021.05.011	20	0.01	2021

### 3.4 Keyword analysis

#### 3.4.1 Keyword Co-occurrence analysis

**Figure 5:**
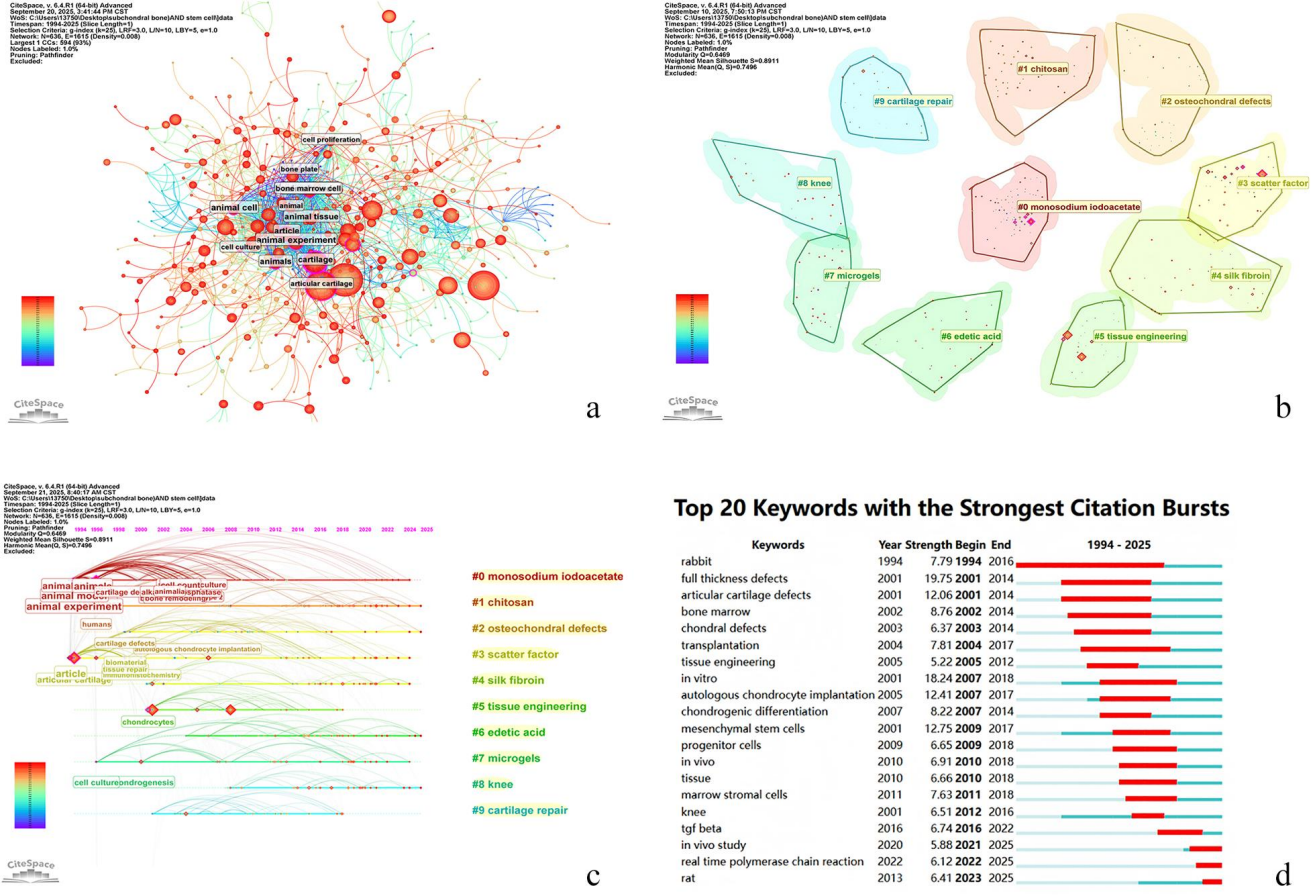
**(a)**: Knowledge map of keywords co-occurrence. **(b)**: Knowledge map of keywords clusters. **(c)**: Timeline view of keywords. **(d)**: Top 20 keywords with the strongest citation bursts.

Keyword co-occurrence analysis generated a network comprising 636 nodes and 1,615 links Figure 5a). Among the top 10 keywords by frequency (Tables 7, 8), “mesenchymal stem cells” ranked first (514 occurrences, centrality 0.02), followed by “articular cartilage” (433 occurrences). The keyword with the highest centrality was “cartilage” (0.27), followed by “animal experiment” (0.17).

**Table 7:** The top 10 highly cited keywords.

Serial number	Cited keyword	Count	Centrality	Year
1	mesenchymal stem cells	514	0.02	2001
2	articular cartilage	433	0.12	1994
3	subchondral bone	397	0.01	2008
4	repair	215	0.01	2001
5	cartilage	213	0.27	1996
6	stem cells	209	0.03	1996
7	article	168	0.16	1994
8	differentiation	154	0.01	2004
9	bone	152	0.03	2002
10	tissue engineering	152	0.01	2005

**Table 8:** The top 10 keywords of centrality.

Serial number	Keyword	Count	Centrality	Year
1	cartilage	213	0.27	1996
2	animal experiment	93	0.17	1994
3	article	168	0.16	1994
4	animal model	89	0.16	1994
5	animals	70	0.15	1996
6	cell culture	23	0.15	1996
7	chondrocytes	117	0.13	2001
8	articular cartilage	433	0.12	1994
9	animal cell	65	0.11	1994
10	full thickness defects	48	0.11	2001

#### 3.4.2 Keyword clustering analysis

Keyword clustering was performed using the LLR algorithm, resulting in 10 clusters. The cluster map (Figure 5b) showed a Modularity Q of 0.6469 (>0.3) and a Mean Silhouette of 0.8911 (>0.7), indicating a significant and credible clustering structure. The 10 clusters were: #0 monosodium iodoacetate, #1 chitosan, #2 osteochondral defects, #3 scatter factor, #4 silk fibroin, #5 tissue engineering, #6 edetic acid, #7 microgels, #8 knee, and #9 cartilage repair. The main contents of each cluster are shown in Table 9, and the corresponding timeline view is presented in Figure 5c. The ten keyword clusters in the field of cartilage injury and stem cells form a complete translational pipeline—from basic research to clinical application—through a progressive synergy of mechanisms, technologies, and clinical strategies. Their clinical significance lies in offering an innovative therapy capable of restoring cartilage structure and delaying disease progression. Future efforts should focus more on interdisciplinary collaboration and standardization to accelerate translation from bench to bedside.

**Table 9:** The top 10 clustering tables of keywords.

Cluster	Size	Silhouette	Year	Label (LLR)
0	87	0.866	2004	monosodium iodoacetate; monocyte chemotactic protein 1; cartilage cell; microfracture; autologous chondrocyte implantation
1	50	0.896	2016	chitosan; fluorescence activated cell sorting; drug efficacy; adipose-derived mesenchymal stem cell; regenerative medicine
2	46	0.881	2009	osteochondral defects; autologous chondrocyte implantation; stromal cells; microfracture; model
3	43	0.899	2008	scatter factor; interleukin 8; transforming growth factor; article; 3d printing
4	43	0.885	2014	silk fibroin; osteochondral regeneration; allografts; transcriptomics; postoperative care
5	37	0.884	2009	tissue engineering; mesenchymal stem cells; calcified cartilage; vasculotropin; chondroitin
6	35	0.797	2016	edetic acid; temporomandibular joint; cell culture techniques; tgf beta; hydroxyapatite
7	34	0.91	2013	microgels; temperature; vasculotropin a; chondrogenesis; cell separation
8	31	0.931	2016	knee; autophagy; cycloheximide; skeleton; freeze drying
9	31	0.898	2010	cartilage repair; differentiation; articular cartilage repair; microfracture; tissue

#### 3.4.3 Analysis of keyword bursts

Burst detection identified 25 keywords, with the top 20 strongest bursts shown in Figure 5d. The “Strength” indicates burst intensity; “Begin” and “End” mark the start and end of the burst period, corresponding to the red segments in the figure. The keyword burst graph displays the top 20 keywords with the strongest citation bursts during the period from 1994 to 2025 (Figure 5d). These keywords with citation bursts indicate they received significant attention during specific time periods. The chart shows that these citation bursts were concentrated in two main periods: 2001–2018 and the recent period, particularly 2020–2025.

Early research hotspots (2001–2018) primarily focused on animal models and experiments, cartilage and bone defects, cell therapy and tissue engineering, as well as transplantation and *in vitro* studies. The keyword “rabbit,” referring to an experimental animal model, exhibited a significant citation burst as early as 1994, demonstrating the importance of animal models in early research. Meanwhile, terms such as “full thickness defects,” “articular cartilage defects,” “bone marrow,” and “chondral defects” also showed notable citation bursts, indicating that cartilage and bone repair and regeneration were key research focuses during this period (31, 32). Keywords such as “mesenchymal stem cells,” “progenitor cells,” and “tissue engineering” exhibited notable citation bursts, reflecting the emergence of cell therapy and tissue engineering in the field of cartilage and stem cell regenerative medicine (33). The citation bursts of keywords such as “transplantation” and “*in vitro*” underscore the importance of transplantation techniques and *in vitro* studies in medical research.

More recently (2020–2025), the field has not so much shifted toward *in vivo* work as it has deepened its focus on it, with emerging bursts in “*in vivo* study” and “real-time polymerase chain reaction”. The citation bursts of keywords such as “*in vivo* study” and “real-time polymerase chain reaction” reflect a growing emphasis on animal models and molecular biology techniques to elucidate the regulatory mechanisms of skeletal stem cells and their fate determination (34, 35). The citation burst of keywords such as “tgf beta” reflects researchers’ in-depth exploration of the roles played by specific factors and signaling pathways in biological processes within cartilage and stem cell studies. It is widely recognized that aberrant activation or inhibition of multiple signaling pathways contributes to pathological changes in temporomandibular joint osteoarthritis tissues, promoting cartilage matrix degradation, catabolism, and chondrocyte apoptosis (36).

The evolution of research hotspots indicates that advances in novel real-time imaging technologies, breakthroughs in molecular biology techniques—such as single-cell sequencing and gene editing—and the cumulative impact of earlier foundational studies have collectively shifted recent research emphasis toward *in vivo* investigations, real-time monitoring approaches, and the roles of specific molecular factors and signaling pathways.

## 4 Discussion

### 4.1 Research Status and development trends

This study indicates that from 1994 to 2005, the number of publications in this field showed an overall steady upward trend, which can be divided into four stages and three peaks.

Stage 1: From 1994 to 2000, the number of publications developed with fluctuations. During this period, research remained in an initial exploratory phase, primarily focused on fundamental scientific inquiries aimed at evaluating the feasibility of stem cells in cartilage repair. Due to the immaturity of relevant technologies and the lack of a cohesive research framework, studies were fragmented, resulting in considerable fluctuations in publication output.

Stage 2: From 2001 to 2010, the number of publications increased year by year. With the rise of stem cell research and the widespread adoption of concepts in tissue engineering and regenerative medicine, this field gradually emerged as a research hotspot. Key technological advances during this phase—including *in vitro* expansion and directed differentiation of stem cells, development of scaffold biomaterials, and early-stage validation studies in animal models—likely served as primary drivers behind the steady increase in publication output.

Stage 3: From 2012 to 2021, the number of publications increased significantly, showing a rapid development trend, with a peak in 2021 (123 publications). During this period, significant advances were achieved through the integration of stem cell technology with bioengineering approaches. Research focus shifted toward emerging topics such as exosomes, bioactive scaffolds, and novel signaling modulation mechanisms. The peak in publication output observed in 2021 can be attributed to several interrelated factors: the efficient incorporation of smart medical technologies—including single-cell sequencing and high-throughput screening—the breakthrough development of advanced biomaterials (e.g., functionalized hydrogels), and the adoption of more sophisticated experimental models, such as three-dimensional *in vitro* culture systems and large-animal models. Additionally, intensified collaboration between academia and industry likely further accelerated research productivity and innovation during this phase.

Stage 4: Although the number of publications declined from 2023 to 2025, it remained at a high level. This shift suggests a transition from purely fundamental inquiry toward clinical translation and precision therapeutics—evidenced by an increasing focus on personalized interventions for specific pathologies such as osteoarthritis and the re-evaluation of current clinical guidelines. The field now prioritizes high-quality, clinically meaningful innovations over sheer publication volume. Consistent with this trajectory, a linear regression model (Y = 4.2163X−8433.4) projects a continued upward trend in future publication output. Projections based on the linear function formula (Y = 4.2163X-8433.4) also indicate a continuing upward trend in future publication output.

These findings demonstrate that research on the relationship between subchondral bone and stem cells represents an emerging focus with promising prospects and substantial potential for further development.

The most prolific authors were Zhang Wei (15 publications, accounting for 1.18%) and Yao Qingqiang (14 publications, 1.1%), who belong to the same research group. Their main research focuses on applying composite hydrogel scaffolds (37, 38) and 3D-printed composite bio-scaffolds for tissue-engineered bone studies (39, 40) to repair cartilage and subchondral bone injuries. However, we also observed generally low centrality among authors in this field, with no author having a centrality>0.1, suggesting that research in this area may still be in an exploratory stage. Chinese Academy of Sciencespublished the most articles (52) and demonstrated relatively strong centrality (0.19). This is also the affiliated institution of Zhang Wei and Yao Qingqiang, indicating that Chinese institutions play a prominent and collaborative role in this field. The Chinese Academy of Sciences and Zhejiang University ranked first and second, respectively, in both publication volume and centrality, further confirming the core status of Chinese institutions in this research field. While China and the United States lead in publication volume, ranking first and second respectively, Japan-ranked fourth in output-demonstrated the highest centrality (0.7). This indicates that Japan holds a pivotal role in the research network, whereas China and the U.S. may need to enhance research quality, increase consensus within the field, and strengthen international collaboration. Among the top 10 countries/regions by publication volume, the majority are from Asia (PEOPLES R CHINA, Japan, South Korea), North America (USA, Canada), Europe (Germany, Italy, Spain, Switzerland, France), and Oceania (Australia). The presence of multiple European countries indicates a broader regional engagement in this field of research.​

Analysis of cited authors shows that ZHEN GH had the highest citation count but low centrality (0.03). His team primarily focuses on the role of TGF-*β* signaling pathways and angiogenesis in subchondral bone to mitigate osteoarthritis progression (41–43). Authors ranked third (BRITTBERG M) and ninth (BUCKWALTER JA) achieved a centrality of 0.16, indicating their research holds greater structural influence in the field. This suggests Chinese research teams should enhance their core competitiveness to achieve broader academic recognition.​The Dutch journal ACTA ORTHOP SCAND the American journal AM J SPORT MED demonstrated the highest centrality values (0.34 and 0.31, respectively). Notably, The American Journal of Sports Medicinewas cited over 300 times and is recognized as a high-impact journal (Q1, TOP journal), underscoring the authority and significant influence of these two publications within this research field. Zhen GH's article received the highest number of citations, primarily for revealing that high concentrations of TGF-*β*1 in the subchondral bone may be a critical pathological factor in triggering osteoarthritis. Thus, inhibiting this process could represent a potential therapeutic strategy for the disease (43). Three co-cited references were published in Acta Biomater, further indicating the persuasive impact of high-impact journals. Among them, Levingstone TJ et al. (44, 45) developed a multi-layered collagen scaffold to repair subchondral bone in rabbit knee joints, demonstrating its ability to mobilize the self-renewal potential of host stem cells in the injured area, promote subchondral bone repair, and achieve tissue regeneration.

Keyword co-occurrence analysis identified “articular cartilage,” “repair,” “cartilage,” and “animal experiment” as prominent keywords in this field. Interestingly, our findings also revealed a notable discrepancy between frequency and centrality: while “mesenchymal stem cells” appeared most frequently (514 occurrences), they exhibited a low centrality score (0.02); in contrast, “cartilage” demonstrated the highest centrality (0.27). We speculate that although mesenchymal stem cells are frequently mentioned, they are often used in a standardized and modular context, and thus do not function as a bridge linking different scientific domains. In comparison, “cartilage” shows the highest centrality, suggesting it serves as a core scientific issue that integrates diverse areas of knowledge. This phenomenon reflects an underlying structural tension between fundamental research and application-oriented demands. Addressing this issue requires a stronger focus on clinical value, along with efforts to promote interdisciplinary collaboration and support industrial translation. Only through such integration can the widespread academic interest in “stem cells” be channeled into tangible therapeutic outcomes for “cartilage repair,” ultimately advancing both scientific impact and patient benefit.

Clustering analysis identified multiple research hotspots in the cartilage and stem cell field, with each cluster representing a distinct research direction or thematic collection. Analysis identified the following primary categories: 1. Cartilage Repair and Regenerative Medicine: For example, Cluster 0 includes keywords such as “monosodium iodoacetate,” “monocyte chemotactic protein 1,” and “cartilage cells,” indicating that chondrocyte damage and repair represent a significant research focus. Furthermore, the frequent mention of “autologous chondrocyte implantation” and “microfracture” further underscores the prominence of cartilage repair techniques as a key research focus. Similarly, Clusters 2 and 9 focus on “osteochondral defects” and “articular cartilage repair,” respectively, reflecting sustained research interest in cartilage regeneration. 2. Regenerative Medicine and Tissue Engineering: Cluster 1, containing keywords such as “chitosan” and “adipose-derived mesenchymal stem cell,” reflects the combined application of biomaterials and stem cell technology in regenerative medicine. Cluster 5, focusing on “tissue engineering” and “mesenchymal stem cells,” highlights its central role in regenerative medicine. 3. Cytokines and Growth Factors: Cluster 3 includes keywords such as “scatter factor,” “interleukin 8,” and “transforming growth factor,” highlighting the significance of cytokines in the regulation of biological processes. 4. Biomaterials and 3D Printing Technology: Cluster 4 references “silk fibroin” and “3D printing,” reflecting the application of biomaterials in 3D printing technology, particularly their potential in tissue engineering and regenerative medicine. Cluster 7, involving “microgels” and “cell separation,” demonstrates the use of microscale biomaterials in cell manipulation and separation. 5. Skeletal System and Autophagy Mechanisms: Cluster 8 focuses on keywords such as “knee” and “autophagy,” revealing the role of autophagy mechanisms in cartilage and stem cell research. According to the timeline view of clusters, stem cells (46), differentiation (47), regeneration (48), and cartilage repair (49) will remain key research hotspots in the future.

Analysis of keywords with the strongest citation bursts reveals that cartilage and bone repair, cell therapy and tissue engineering, animal models and experiments, as well as transplantation and *in vitro* studies have been consistent research hotspots. Concurrently, with technological advances and deeper investigation, *in vivo* studies, real-time monitoring techniques, and research on specific factors and signaling pathways are gradually emerging as new focuse (50–53).

The bibliometric findings of this study offer clear guidance for clinical practice. First, the persistent focus on “tissue engineering” and “cartilage/bone repair” suggests that future joint-preserving or arthroplasty procedures should prioritize biphasic scaffold materials capable of simultaneously regenerating both cartilage and subchondral bone. Second, the sustained interest in signaling pathways like TGF-*β* implies that clinical research could explore these molecules as biomarkers for postoperative osseointegration to enable personalized treatment. Furthermore, the rising prominence of MSCs and their exosomes points toward a promising shift toward “cell-free” biotherapies, which could simplify surgical protocols and enhance safety. Together, these trends point to a future of more precise, integrated, and effective orthopedic regenerative strategies.

### 4.2 Conclusions and implications

This study analyzed publications on the relationship between subchondral bone and stem cells from 1994 to 2025, retrieved from three major databases: WOSCC, PubMed, and Scopus. Using CiteSpace, a bibliometric and visual analysis was conducted, revealing that research on the subchondral bone–stem cell relationship is an emerging academic focus. Key research areas include cartilage and bone repair, cell therapy and tissue engineering, animal models and experiments, as well as transplantation and *in vitro* studies. This study extends beyond the scope of Wen Shuaibo's research, which primarily focused on technological advancements in cartilage repair (47). By contrast, we propose a three-dimensional future framework integrating mechanistic insights (deciphering interface signaling pathways such as TGF-*β*), technological innovations (development of engineered exosomes and smart 3D bioprinted scaffolds), and ecological strategies (deepening global collaboration for high-output nations). This approach not only advances mechanistic understanding of cartilage repair but also provides strategic guidance from a research ecology perspective, addressing a critical gap in bibliometric analyses of global collaboration networks and establishing a systematic roadmap for clinical translation. While China has made substantial contributions in this field, enhancing core competitiveness remains essential. Collaboration among countries, institutions, and researchers should be strengthened to advance exploration in this domain. This study may help researchers understand current trends, hotspots, and developments in the field, and serve as a reference for future investigations.

Our findings closely align with Yang et al.'s (54) bibliometric study on stem cells for meniscal regeneration and 3D printing in cartilage repair. Both identify “tissue engineering,” “mesenchymal stem cells,” and “3D printing” as dominant research fronts, reflecting a shared paradigm of combining biomaterials and stem cells for tissue reconstruction. The prominence of biomaterials like “silk fibroin” (Cluster #4) further corroborates their observations.

However, key differences arise from distinct anatomical focuses. Yang et al. emphasize the meniscus—a fibrocartilaginous, load-bearing structure—highlighting mechanical integration with ligaments. In contrast, our analysis of the subchondral bone–cartilage unit reveals unique hotspots centered on bone-cartilage crosstalk, particularly TGF-*β* signaling (Cluster #3) and subchondral-specific pathologies (e.g., monosodium iodoacetate models, Cluster #0). This indicates that while core technologies are shared across joint regeneration fields, therapeutic strategies are tailored to each tissue's specific microenvironment and functional demands.

## 5 Limitations

This study provides a bibliometric overview of research on subchondral bone and stem cells; however, several limitations should be acknowledged.

First, we restricted our analysis to English-language articles and reviews from Web of Science, Scopus, and PubMed. Given China's dominant contribution (45.22% of publications), this likely excluded relevant high quality studies published in Chinese journals introducing potential language bias and underrepresenting regional research perspectives.

Second, excluding other document types may have omitted emerging ideas or clinical insights. Third, bibliometric tools like CiteSpace and VOSviewer cannot evaluate study quality—highly cited but methodologically weak work may thus unduly influence results. Finally, keyword-based analyses depend on author-provided terms, which can be inconsistent or incomplete, potentially affecting thematic accuracy.

## 6 Perspective

Future research should focus on elucidating subchondral bone–cartilage crosstalk, advancing cell-free therapies (e.g., engineered exosomes), and developing smart, 3D-bioprinted scaffolds. Validation in large-animal models and robust clinical trials is essential. Integration of single-cell omics, organoids, and AI will drive mechanistic discovery, while enhanced global collaborations is key to reducing bias and accelerating clinical translation.

## Data Availability

The original contributions presented in the study are included in the article/supplementary material, further inquiries can be directed to the corresponding authors.
